# Personalized gait trajectory generation for lower-limb rehabilitation robots using anthropometric features

**DOI:** 10.1016/j.isci.2026.116958

**Published:** 2026-08-06

**Authors:** Liangjie Tu, Shuai Zhao, Xinyi Tang, Rong Chen, Quanying Liu, Huihui Sun

**Affiliations:** 1 College of Mechanical and Electrical Engineering, Huainan Normal University, Anhui 232038, Huainan, China; 2 Human-computer Collaborative Robot Joint Laboratory of Anhui Province, Huainan 232038, China; 3 College of Mechanical Engineering, Zhejiang University of Technology, Zhejiang 310023, Hangzhou, China

**Keywords:** personalized gait trajectory, improved gaussian process regression model, IGPR, human-robot coordinated motion control, lower-limb rehabilitation robots

## Abstract

Conventional gait generation schemes fail to accommodate variable walking speeds and diverse rehabilitation scenarios. This study constructs an improved Gaussian process regression (IGPR) model with hybrid kernel functions based on 12 anthropometric indicators, walking speed and training modes. The dataset consists of 42 healthy participants, with averaged hip joint angle trajectories *RMSE* = 3.31° and knee joint angle trajectories *RMSE* = 4.37°. We separate model prediction, offline gait generation and robot tracking verification. Tracking tests on four healthy volunteers prove stable trajectory following. This method supplies quantitative gait references for wearable devices, further clinical trials with stroke patients are required to verify rehabilitation outcomes.

## Highlights

• A hybrid-kernel IGPR model is proposed for personalized gait trajectory generation
• A personalized gait method adopts 12 anthropometric features for gait generation
• It generates customized gait trajectories for various speeds and training scenarios

## Graphical abstract

**Figure:**
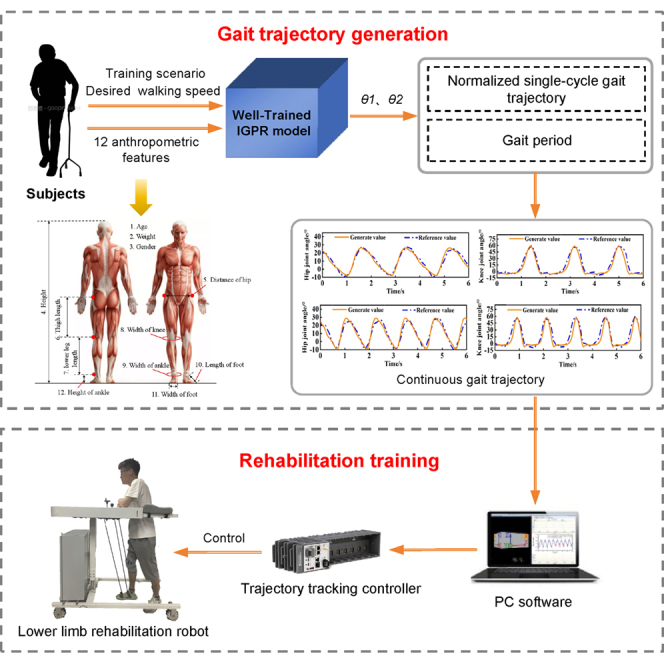

## Teaser

Mechanobiology; Biomechanics; Computational mechanics

## Introduction

Personalized gait trajectory generation is a crucial task for lower limb rehabilitation robots and exoskeleton robots to assist stroke patients to achieve human-robot coordinated movement.^1^ Indeed, individual variations in height, weight, body proportions, and walking habits result in unique gait patterns for each person. Ill-fitting gait trajectories can severely compromise the compliance of human-robot coordinated movement, potentially leading to human-robot opposition and reduced rehabilitation efficiency.^2^ Therefore, generating highly personalized gait trajectories for individual patients is crucial to addressing the challenges mentioned above.

Conventional methods for generating gait trajectories in rehabilitation robotics primarily fall into two categories. The first category is the gait generation method based on predefined joint trajectory, which mainly collects the gait trajectory of healthy people walking on the flat ground by using motion capture system or angle sensors assembled by exoskeleton joints, and constructs gait database after data preprocessing. Then, the gait trajectory of a human with similar anthropometric features (e.g., height, weight) to the patient was selected as the reference gait trajectory for robot tracking during the rehabilitation training of the patient. Examples of lower-limb rehabilitation systems adopting this method include ReWalk,^3^ early versions of eLegs,^4^ and ROBIN.^5^ The second category is based on kinematic and dynamic models. This methodology mainly uses the dynamic and kinematic models^6^^,^^7^^,^^8^ of the human body to calculate the angle trajectory of each joint of the lower limbs during human walking, and then generates the corresponding gait trajectory. For instance, William et al.^9^ proposed a minimum-jerk gait planning method using the Mina exoskeleton robot to generate the spatial trajectory of the ankle joint, and then solved the angle of each joint through the inverse dynamic model to generate the corresponding gait trajectory. The above method was also adopted by Murnmolo et al.^10^ to generate the gait trajectory of the robot in Mina V2. Daniel et al.^11^ proposed a gait trajectory generation method with adjustable step length and step height by constructing an inverted pendulum model of the human body. The above two categories of methods can generate gait trajectories of the lower limb rehabilitation robot, but due to individual differences, the gait trajectories generated by the above methods are difficult to be highly suitable for each patient. Therefore, there is still a problem of insufficient degree of personalization. In addition to the above conventional gait generation methods, dynamic movement primitive and artificial potential field are widely used in robot trajectory planning, which are capable of generating flexible trajectories and realizing dynamic obstacle avoidance.^12^ Moreover, high-precision adaptive human-robot interaction torque estimation has become a key supporting technology for lower-limb rehabilitation robots, which can effectively improve the performance of adaptive gait training and human-robot coordination.^13^ However, most of the above methods focus on trajectory optimization or interactive signal perception, and seldom combine individual anthropometric features to achieve fully personalized gait trajectory generation for different users.

In response to the limitations of the approaches mentioned above, researchers have begun to incorporate hemiplegic patients’ own physiological and motion information into the gait trajectory generation process for lower-limb rehabilitation robots.^14^^,^^15^ For example, Zhang et al.^16^ utilized a BP neural network to establish a mapping relationship between sEMG signals and lower-limb joint angles, enabling continuous gait trajectory generation. Lu et al.^17^ used an inertial measurement unit (IMU) to measure hip joint angles in real time during lower-limb movement, detecting four gait events defined based on hip joint kinematics, and generated corresponding gait trajectories according to these detected events. Although these methods improve the personalized degree of gait trajectory to a certain extent, due to the compensatory mechanism of the healthy leg of stroke patients, the physiological information (e.g., sEMG signals) and motion information (e.g., foot pressure, joint angles) during walking of stroke patients are quite different from those of healthy people.^18^ Therefore, the gait trajectories generated based on the above methods are still difficult to meet the actual rehabilitation needs of stroke patients. In recent years, researchers have further incorporated anthropometric features of stroke patients into gait trajectory generation. For instance, Ren et al.^19^ proposed a gait generation method based on a random forest algorithm, which uses 14 anthropometric features (e.g., foot length, foot width) as input variables to train the model, and reconstructs lower-limb joint angle trajectories via Fourier series to produce the resulting gait. Similarly, Yun et al.^20^ studied the joint angle fitting algorithm based on Gaussian process regression, constructed the database by measuring the lower-limb anthropometric features parameters of the wearer, and constructed the mapping relationship between these anthropometric features and gait trajectories based on the Gaussian process regression algorithm, so as to realize the regression fitting of the lower limb joint angle trajectory. Although the above methods can generate gait trajectories for different stroke patients, and improve the degree of personalization of gait trajectories to a certain extent. However, the effect of walking speed on gait trajectories is not fully considered, and the corresponding gait trajectories cannot be generated according to the actual gait speed needs of patients, which will have a negative impact on the compliance of human-machine coordinated movement control during gait rehabilitation training. In addition, the above method is only suitable for robot-assisted patients to walk at a constant speed on a treadmill, and the scene is single, which is difficult to meet the needs of the actual gait rehabilitation training scenario.

Therefore, this study proposes a novel method for generating personalized gait trajectories based on anthropometric features and an improved Gaussian process regression (IGPR) model. The proposed approach is capable of producing individualized gait trajectories for lower-limb rehabilitation robots across multiple walking speeds and diverse training scenarios. The proposed method introduces an IGPR algorithm. This algorithm is used to construct a mapping model that relates anthropometric features (*P*), desired walking speed (*V*), and training scenario (*G*) to both a normalized single-cycle gait trajectory and its corresponding gait period. Based on this model, by inputting the patient’s anthropometric features (e.g., height, weight) along with the target walking speed and training scenario specified by a rehabilitation therapist, the method can generate a highly personalized gait trajectory that is optimally adapted to each individual patient. The method provides technical support for lower-limb rehabilitation robots to assist stroke patients in performing walking rehabilitation tasks across multiple speeds and various scenarios.

## Results

### Gait trajectory generation result of the IGPR model

**Figure 1:**
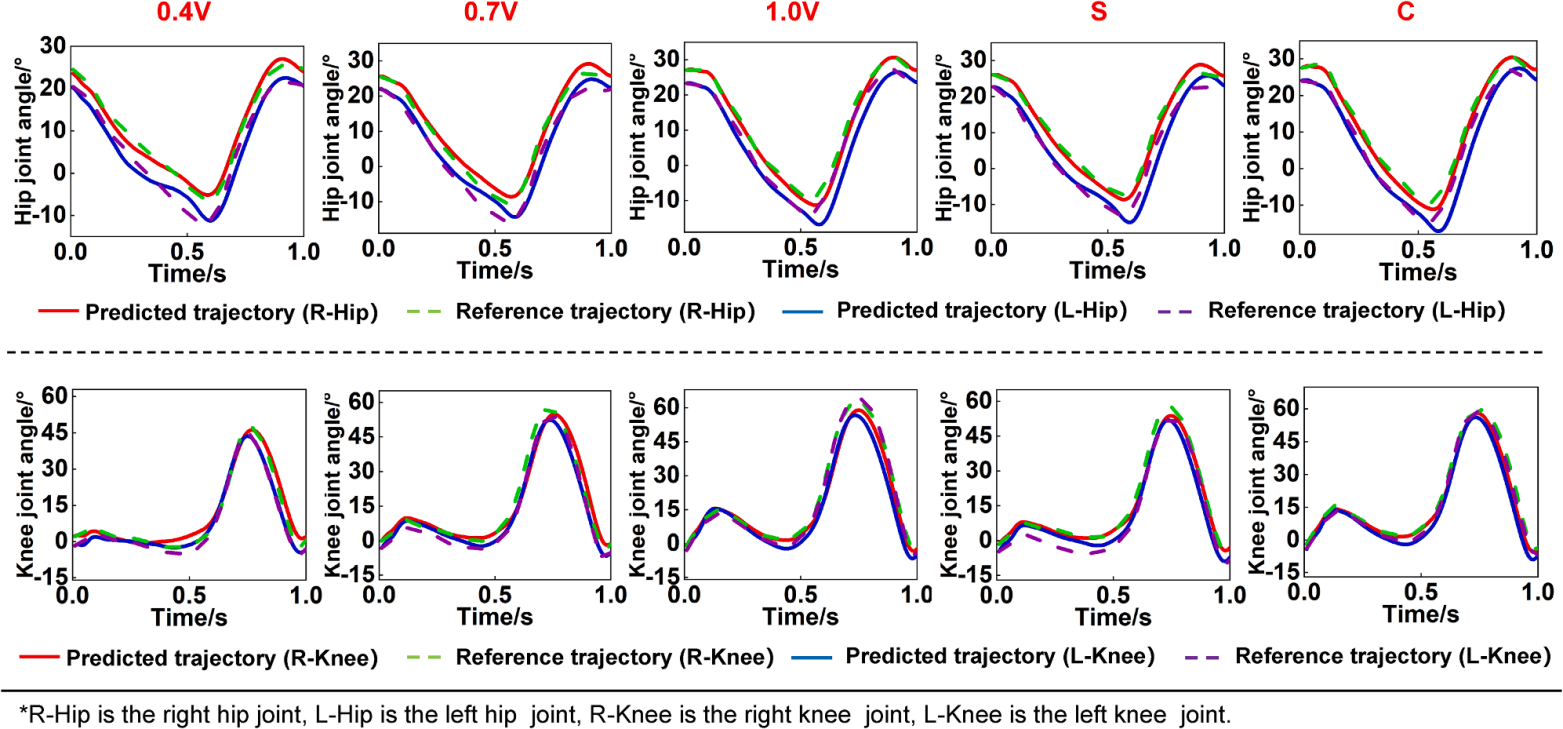
Generated personalized normalized single-cycle gait trajectories for subject S1

**Figure 2:**
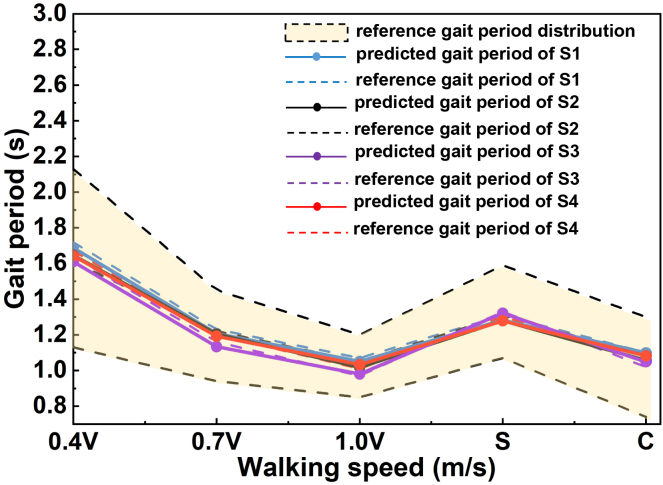
Generated standardized gait periods for the four test subjects across different scenarios and walking speeds

**Figure 3:**
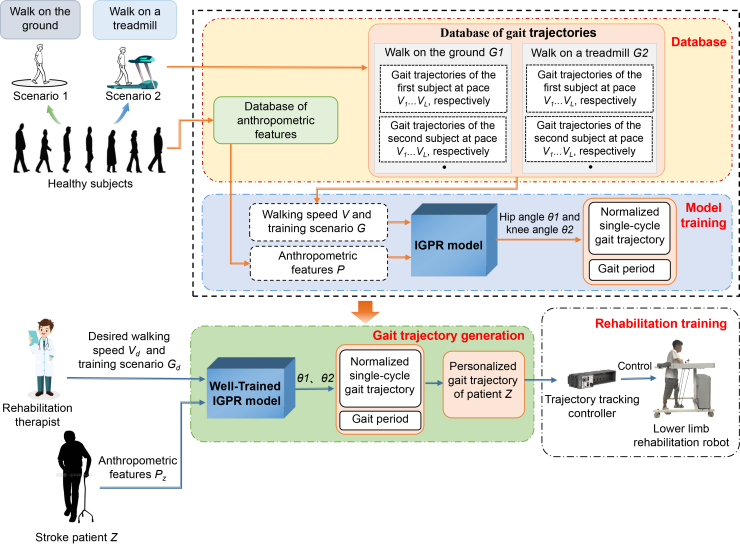
Overall flowchart of the personalized gait trajectory generation method

**Figure 4:**
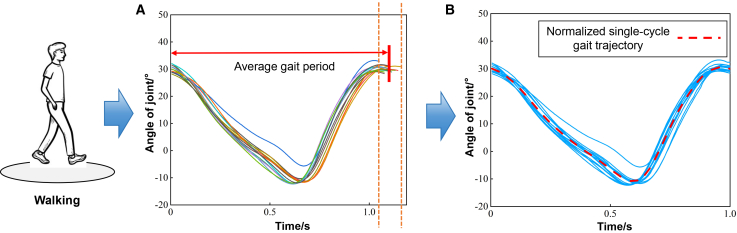
Flowchart of gait trajectory preprocessing (A) Segmented raw single-cycle gait trajectories. (B) Preprocessed normalized single-cycle gait trajectories.

**Figure 5:**
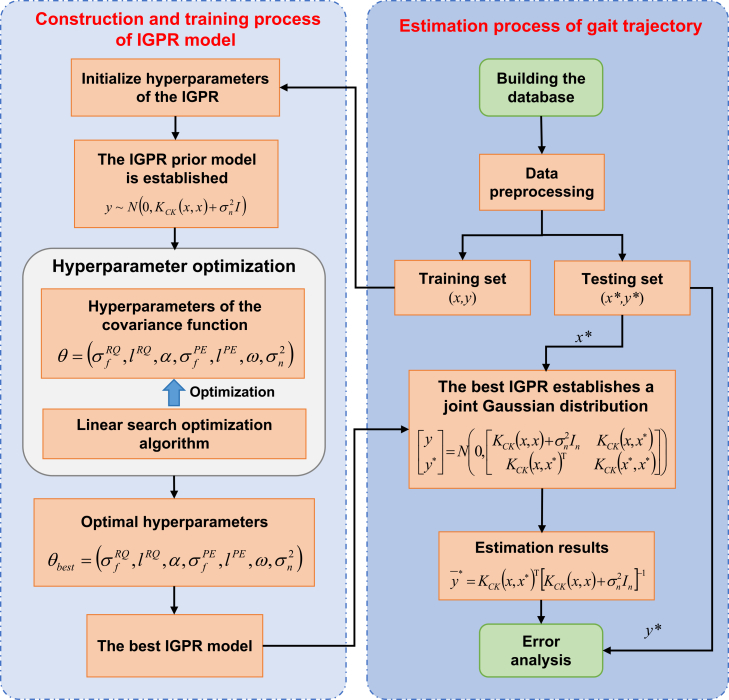
Implementation flowchart of the IGPR model

**Figure 6:**
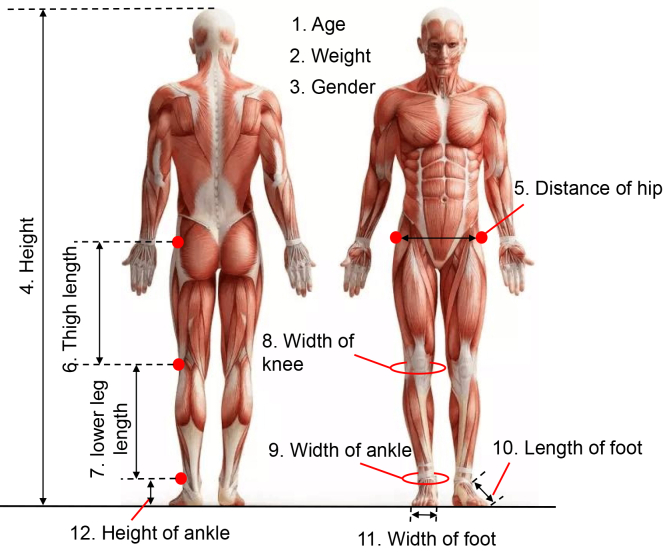
Schematic diagram of anthropometric features

**Figure 7:**
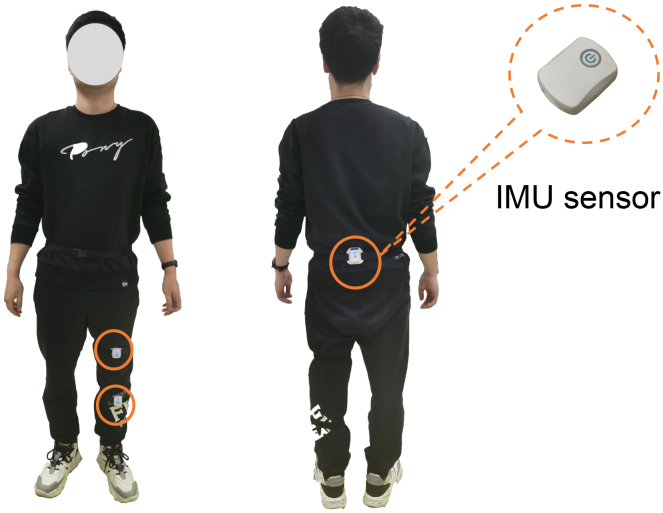
IMU sensor wearing scheme

The ranking of feature importance obtained via mRMR screening is provided in Table 1, which guides the selection of input anthropometric indicators for subsequent model training. The test set was constructed using experimental data from two younger and two older adults within the established dataset, with the remaining 38 subjects’ data forming the training set. Based on this partition, we evaluated the predictive performance of the IGPR model for human gait trajectories. The IGPR model predicts the left and right lower-limb joints independently, because individual left and right lower limbs may have slight differences in anthropometric features and motion patterns. Independent prediction helps improve the personalization and accuracy of bilateral joint trajectories. The walking speeds for the four test subjects—including treadmill speeds (0.4*V* m/s, 0.7*V* m/s, 1.0 *V* m/s) and overground speeds (*S* m/s, *C* m/s)—are listed in Table 2. Figure 1 illustrates the generated personalized normalized single-cycle gait trajectories for subject S1 under different scenarios and walking speeds, obtained using the trained IGPR model. To quantitatively evaluate the prediction accuracy of the IGPR model, this study introduced two evaluation metrics: root-mean-square error (*RMSE*) and absolute error (*AE*), to analyze the gait trajectory generation results for all subjects in the test set. Table 3 presents the *RMSE* values of the generated personalized normalized single-cycle gait trajectories for all test subjects across different scenarios and walking speeds. Figure 2 displays the corresponding standardized gait periods generated for all test subjects under varying conditions. Table 4 lists the *AE*s for these generated gait periods. The full implementation workflow covering dataset construction, model training, trajectory generation and robot validation is visualized in Figures 3, 4, 5, 6, and 7, with detailed schematic diagrams available in the STAR Methods section.

**Table 1:** Importance scores and rankings of 12 anthropometric features based on mRMR

Anthropometric features	Importance score	Ranking
Distance of hip (cm)	0.912	1
Thigh length (cm)	0.857	2
Lower leg length (cm)	0.803	3
Weight (kg)	0.726	4
Height (cm)	0.684	5
Width of knee (cm)	0.591	6
Gender (male/female)	0.502	7
Length of foot (cm)	0.435	8
Height of ankle (cm)	0.387	9
Width of ankle (cm)	0.294	10
Width of foot (cm)	0.231	11
Age (Y/O)	0.156	12

**Table 2:** Walking speeds of the four test subjects under different scenarios

Subject	Treadmill walking speeds(m/s)			Overground walking speeds(m/s)	
	0.4*V*	0.7*V*	1.0*V*	*S*	*C*
S1	0.5	0.9	1.2	0.9	1.2
S2	0.5	0.9	1.3	0.9	1.2
S3	0.4	0.8	1.1	0.7	1.1
S4	0.5	0.9	1.3	0.9	1.3

**Table 3:** *RMSE* of personalized normalized single-cycle gait trajectory predictions for the four test subjects

Speed (m/s)	S1		S2		S3		S4	
	R-H/L-H	R-K/L-K	R-H/L-H	R-K/L-K	R-H/L-H	R-K/L-K	R-H/L-H	R-K/L-K
0.4 *V*	1.24/1.97	2.41/1.55	2.77/2.91	6.58/3.91	3.22/1.74	3.81/3.15	2.58/2.72	3.64/4.21
0.7 *V*	1.57/2.12	3.71/1.93	2.99/2.48	3.14/3.85	3.21/1.67	2.72/3.36	3.47/2.60	4.56/5.57
1.0 *V*	1.63/3.41	3.66/5.57	3.40/4.39	2.56/4.06	2.18/3.29	3.61/3.98	4.36/5.95	3.78/4.15
*S*	1.34/2.44	2.40/3.41	3.58/3.05	6.84/4.61	2.72/1.60	4.25/3.15	2.90/3.44	5.57/3.92
*C*	1.47/2.92	3.12/3.60	3.43/2.97	3.40/5.74	4.20/3.07	3.22/4.29	4.64/3.72	6.17/5.35

Where R-H/L-H denote the *RMSE* of the right/left hip joint trajectories, and R-K/L-K represent the *RMSE* of the right/left knee joint trajectories, respectively.

**Table 4:** Errors in standardized gait period generation for four test subjects(s)

Speed (m/s)	S1	S2	S3	S4
0.4 *V*	0.0316	−0.0360	0.0296	0.0278
0.7 *V*	0.0228	0.0163	0.0268	0.0080
1.0 *V*	0.0197	0.0325	−0.0100	−0.0228
*S*	0.0272	0.0309	0.0088	0.0215
*C*	0.0009	0.0234	−0.0285	0.0078

The results demonstrate that the IGPR model effectively generates normalized single-cycle gait trajectories for different subjects across various scenarios and walking speeds, with both the trend and amplitude of the predicted trajectories showing close agreement with the actual joint angle data (Figure 1; Table 3). Moreover, all generated standardized gait periods fall within the distribution range of the reference cycles, with a maximum AE of only 0.036 s (Figure 2; Table 4). The proposed method therefore offers an effective solution for generating personalized gait trajectories adaptable to multiple scenarios and varying walking speeds.

### The comparison between IGPR and other algorithms

**Figure 8:**
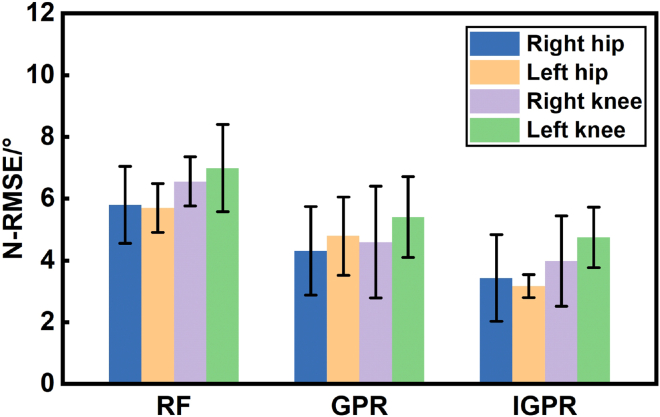
*N-RMSE* of normalized single-cycle gait trajectory generation results based on different models

**Figure 9:**
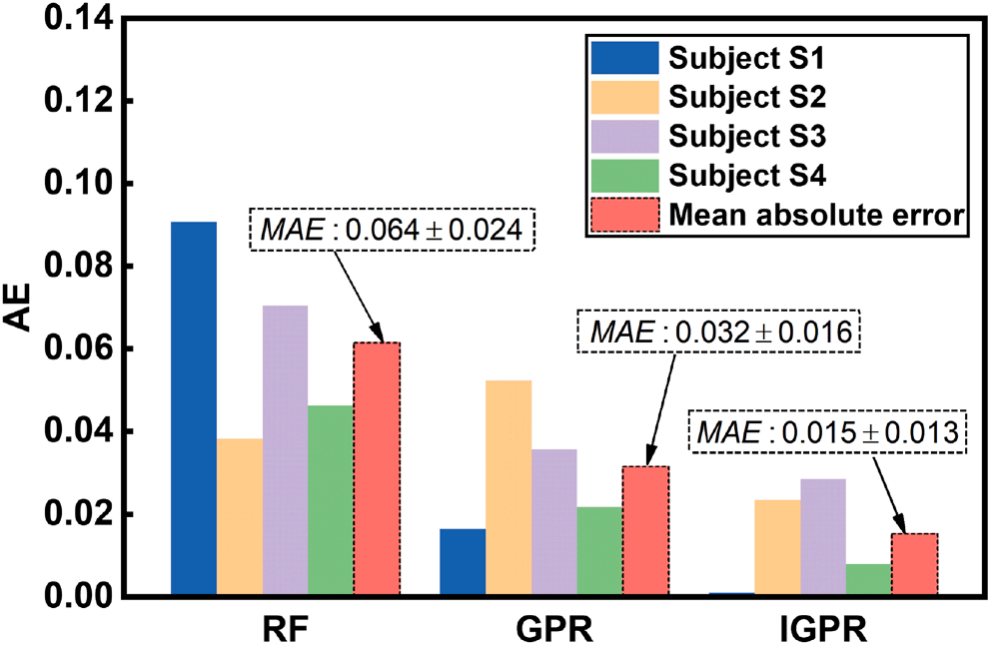
*AE* values of standardized gait period generation results based on different models

To further evaluate the performance of the IGPR model, this study conducted a comparative analysis using the established dataset. While training the proposed IGPR model, we also trained and evaluated two benchmark algorithms on the same dataset: the random forest (RF) algorithm from previous research^19^ and a conventional Gaussian process regression (GPR) algorithm.^21^ To ensure all models were compared under optimal conditions, the linear search algorithm was also applied to optimize the hyperparameters of the conventional GPR algorithm. Table 5 presents the *RMSE* values of the normalized single-cycle gait trajectories generated by the different models for the four test subjects walking at normal pace (*C* m/s) overground. Figure 8 shows the *N-RMSE* (mean *RMSE* ± standard deviation) of all generated normalized single-cycle gait trajectories across the different models. Figure 9 displays the *AEs* in the standardized gait periods generated by the respective models for the four test subjects under the same walking condition (normal pace *C* m/s overground).

**Table 5:** *RMSE* of generated personalized normalized single-cycle gait trajectories for the four test subjects at normal walking speed (*C* m/s)

Subject	RF		GPR		IGPR	
	R-H/L-H	R-K/L-K	R-H/L-H	R-K/L-K	R-H/L-H	R-K/L-K
S1	4.87/4.75	5.67/5.04	2.76/4.04	4.39/5.17	1.47/2.92	3.12/3.60
S2	4.93/5.35	6.34/6.91	4.11/4.38	3.32/4.46	3.43/2.97	3.40/5.74
S3	5.88/6.45	7.59/8.28	4.14/4.06	3.45/4.67	4.20/3.07	3.22/4.29
S4	7.54/6.23	6.63/7.73	6.23/6.67	7.21/7.32	4.64/3.72	6.17/5.35
Average	5.81/5.69	6.56/6.99	4.31/4.79	4.59/5.41	3.44/3.17	3.98/4.75

Where R-H/L-H denote the *RMSE* of the right/left hip joint trajectories, and R-K/L-K represent the *RMSE* of the right/left knee joint trajectories, respectively.

Table 5 summarizes the *RMSE* results of different models for the four test subjects under generalization conditions. A comparison of the *RMSE* values for personalized gait trajectories reveals that while all models demonstrate satisfactory performance for some subjects, the IGPR model achieves higher overall accuracy and superior error stability. As shown in Figure 8, the IGPR model exhibits lower mean *RMSE* and standard deviation (*N-RMSE*) compared to the other models, indicating stronger generalization capability and reduced inter-subject error variability. Furthermore, Figure 9 demonstrates that the IGPR model achieves a significantly lower mean AE (*MAE*), confirming its enhanced stability and superior output precision.

### Comparative verification and computational efficiency analysis

To further verify the superiority of the proposed hybrid kernel function, the effectiveness of the hyperparameter optimization strategy, and the real-time performance of the model in applications, comparative experiments on kernel functions, hyperparameter optimization methods, and computational efficiency analysis were carried out in this section.

First, we conducted comparative experiments with different kernel functions. To verify the effectiveness of the hybrid kernel function (rational quadratic + periodic) proposed in this study, five typical kernel combinations were used for comparative experiments, namely single *RBF* kernel, single rational quadratic kernel, single periodic kernel, *RBF* + periodic kernel, and the hybrid kernel adopted in this study. The prediction errors of bilateral hip and knee joint angle trajectories are shown in Table 6.

**Table 6:** Comparison of gait trajectory prediction errors with different kernel functions

Kernel combination	Right hip	Left hip	Right knee	Left knee	Average *RMSE*
Single *RBF* kernel	4.52°	4.68°	5.86°	6.12°	5.30°
Single rational quadratic kernel	3.15°	4.31°	5.43°	5.67°	4.64°
Single periodic kernel	5.27°	5.41°	6.75°	6.98°	6.10°
*RBF* + periodic kernel	3.36°	3.92°	4.89°	5.11°	4.32°
Rational quadratic + periodic (proposed)	3.44°	3.17°	3.98°	4.75°	3.84°

It can be seen from Table 6 that the single periodic kernel has the largest prediction error because it cannot fit the global trend of gait trajectories effectively. The single rational quadratic kernel and *RBF* kernel can capture the global variation of gait, but they ignore the local periodic characteristics of human walking. The *RBF* + periodic kernel combination improves the fitting performance to a certain extent, but it still cannot balance the global and local features of gait. In contrast, the rational quadratic + periodic hybrid kernel proposed in this study achieves the lowest average *RMSE* of 3.84°, which can simultaneously retain the global trend and local periodic details of lower-limb joint trajectories, and significantly improves the accuracy of personalized gait prediction.

Secondly, we compared different methods for optimizing hyperparameters. To validate the advancement of the linear search algorithm used for hyperparameter optimization in the IGPR model, three optimization methods, namely maximum likelihood estimation (MLE), Bayesian optimization, and linear search, were compared. The comparison results are shown in Table 7.

**Table 7:** Performance comparison of different hyperparameter optimization methods

Optimization method	Average *RMSE* (°)	Iteration count	Training time (s)	Stability
Maximum likelihood estimation (MLE)	4.27	86	12.3	Poor (sensitive to initial value)
Bayesian optimization	4.05	42	18.7	Good
Linear search (proposed)	3.84	35	9.5	Best

As shown in Table 7, the MLE method is sensitive to initial values, requires more iterations, and has poor stability, resulting in relatively high prediction error. Bayesian optimization achieves better accuracy and stability, but it takes longer training time and has lower efficiency. The linear search algorithm adopted in this study obtains the lowest average *RMSE* with the least number of iterations and the shortest training time, and shows the best stability. Therefore, linear search is more suitable for hyperparameter optimization of the IGPR model for personalized gait trajectory generation.

In addition, we conducted an analysis of computational efficiency and real-time performance. Computational efficiency and real-time performance are key indicators for the clinical application of lower-limb rehabilitation robots. The computational efficiency of the proposed IGPR model was tested on a computer with Intel Core i7-10700 processor and 16 GB RAM, and the results are shown in Table 8. It can be seen that the time for single personalized gait trajectory prediction is only 18.6 ms, which is lower than the 50 ms real-time control threshold of lower-limb rehabilitation robots and meets the clinical real-time requirements. The model training for 38 samples is completed within 9.5 s, and the offline training mode is adopted without affecting the efficiency of online prediction. The memory usage of the model is only 128 MB, with low resource occupation. For larger datasets (100 samples), the single prediction time of the standard IGPR model is 42.3 ms, which still meets the real-time demand. In addition, for large-scale data scenarios, sparse Gaussian process regression can be introduced to further reduce computational complexity and improve the scalability of the model.

**Table 8:** Computational efficiency and real-time performance of the IGPR model

Test item	Time cost	Description
Single gait trajectory prediction	18.6 ms	Meet real-time requirement (<50 ms)
Model training (38 samples)	9.5 s	Offline training, no impact on online use
Memory usage	128 MB	Low resource occupation
Scalability (100 samples)	42.3 ms perprediction	Sparse GPR can be used for further acceleration

To further explore the model performance under large-scale datasets, we conducted supplementary simulation tests on a dataset with 500 samples. The core optimization idea of sparse GPR is to select a small number of representative inducing points from the original samples to reconstruct the covariance matrix, so as to reduce matrix dimension and cut down computational load. We compared the prediction performance between standard IGPR and sparse IGPR under the same hardware environment. The simulation results show that compared with the original IGPR, the single prediction time of sparse IGPR is reduced from 86.5 ms to 45.6 ms, while the prediction *RMSE* only increases by 0.61°. The results prove that sparse GPR can effectively reduce computational complexity while maintaining high prediction accuracy, which further verifies the engineering application potential of the proposed method for large-scale data scenarios.

### Application validation result of the algorithm

**Figure 10:**
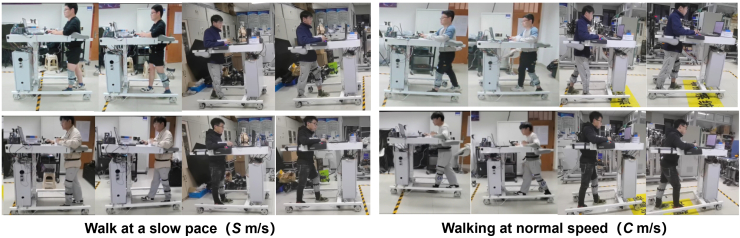
Experiment process of the walking

To further validate the feasibility of the proposed method, an algorithmic application verification was conducted using the self-developed flexible lower-limb rehabilitation robot from our research group. As shown in Figure 10, the flexible lower-limb gait rehabilitation robot assisted 4 subjects (S1, S2, S3, S4) during walking experiments on level ground at two speed modes: slow pace (*S* m/s) and normal pace (*C* m/s). The left leg was designated as the paretic leg, while the right leg served as the healthy leg. The anthropometric features of the two subjects are listed in Table 9.

**Table 9:** Anthropometric features of four subjects

Anthropometric features	S1	S2	S3	S4
Age (Y/O)	31	25	25	30
Weight (kg)	76.0	62.0	65.0	66.0
Height (cm)	173.0	170.5	169.0	175.5
Gender (male/female)	Male	Male	Male	Male
Distance of hip (cm)	39.5	38.5	35.6	35.9
Thigh length (cm)	43.0	47.0	44.3	43.7
lower leg length (cm)	35.5	34.5	32.2	37.4
Width of knee (cm)	12.0	12.5	13.2	11.7
Width of ankle (cm)	8.0	7.5	7.6	7.7
Length of foot (cm)	18.0	17.0	17.8	19.1
Width of foot (cm)	8.0	7.5	7.4	8.5
lower leg length (cm)	35.5	34.5	32.2	37.4

**Figure 11:**
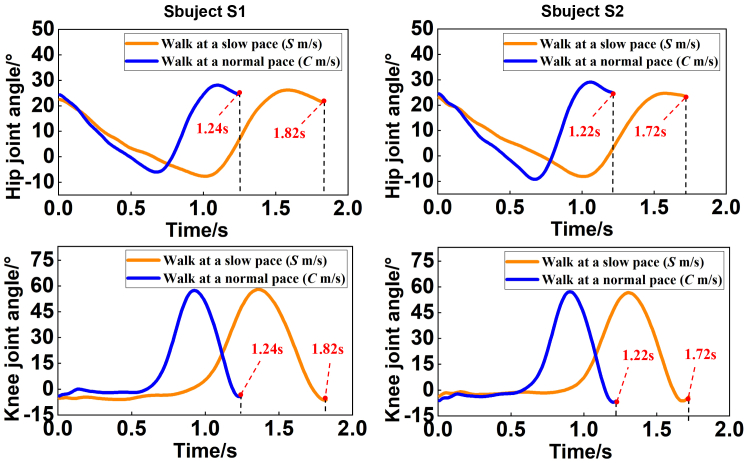
Generated personalized gait trajectories for the paretic leg

**Figure 12:**
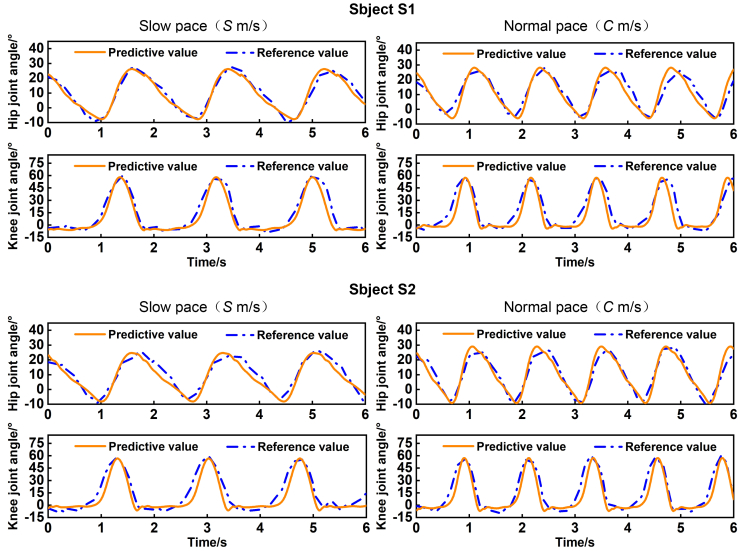
Gait trajectory tracking results of the flexible lower-limb rehabilitation robot under different walking modes

Figure 11 displays the generated personalized gait trajectories for the paretic leg of the representative subjects during slow pace (*S* m/s) and normal pace (*C* m/s) overground walking, obtained using the trained IGPR model. This study defined the initiation of robot controller action as the time-base origin. Figure 12 presents a comparison between the generated personalized trajectories and the actual gait trajectories calculated from IMU data for the representative subjects under different walking modes. The average tracking performance across all four subjects yielded the following results: under slow walking speed, *N-RMSE*_hip_ = 3.13°, *N-R*^2^_hip_ = 0.92 for the hip joint, and *N-RMSE*_knee_ = 5.43°, *N-R*^2^_knee_ = 0.93 for the knee joint; under normal walking speed, *N-RMSE*_hip_ = 4.01°, *N-R*^2^_hip_ = 0.89 for the hip joint, and *N-RMSE*_knee_ = 6.82°, *N-R*^2^_knee_ = 0.90 for the knee joint.

In addition to traditional *RMSE* that reflects fitting accuracy, the coefficient of determination *R*^2^ is adopted as a key metric to evaluate the personalization level of the generated trajectories. A high *R*^2^ value indicates that the predicted gait trajectory is highly consistent with the individual physical characteristics of the subject, which directly reflects the quality of the personalized rehabilitation reference trajectory. The experimental results demonstrate that the proposed personalized gait generation method, based on anthropometric features and the IGPR model, can be effectively applied to lower-limb rehabilitation robotic platforms to generate individualized gait patterns and conduct rehabilitation training for different subjects under various motion modes. From the perspective of gait trajectory tracking, the self-developed flexible lower-limb gait rehabilitation robot with its tracking controller successfully follows the personalized gait trajectories generated by the IGPR model. Although certain tracking errors exist, the results maintain a high degree of fit with the reference trajectories, fully meeting the requirements for gait rehabilitation training.

The observed tracking errors may be attributed to the following two factors. First, vibrations originating from the mechanical structure of the flexible lower-limb gait rehabilitation robot and the rotation of its mecanum wheels during rehabilitation may have introduced measurement noise, leading to computational inaccuracies in the joint angles derived from the IMU sensors attached to the subject’s body. Second, lower-limb inertia during the swing phase likely caused a discrepancy between the actual traction point position and the modeled traction point, resulting in deviations in the computed trajectory.

## Discussion

This study presents a novel method for generating personalized gait trajectories for lower-limb rehabilitation robots, based on anthropometric features and an IGPR model. The proposed approach can effectively generate individualized gait trajectories adapted to different scenarios, walking speeds, and patients—without relying on the patient’s physiological or motion information.

This study selected Gaussian process regression (GPR) as the foundational predictive model for offline generation of personalized gait trajectories due to its distinct advantages over commonly used algorithms such as backpropagation (BP),^22^ random forest (RF),^19^ and long short-term memory.^23^^,^^24^^,^^25^ Specifically, GPR requires fewer tunable parameters, exhibits stronger generalization capability, and provides predictions with probabilistic interpretability. Furthermore, the inherent nonlinear relationship between anthropometric features and gait trajectories—which approximately follows a Gaussian distribution—aligns well with the theoretical foundations of GPR. To enhance the accuracy and generalizability of the generated personalized trajectories, the study further improved the conventional GPR algorithm by constructing a hybrid kernel function, thereby optimizing the model’s predictive performance across different feature scales.

From the perspective of personalized gait trajectory generation, the proposed method successfully achieves offline generation of gait trajectories for different patients across various scenarios and walking speeds. For the four test subjects, the average *RMSE* of the generated bilateral hip joint angle trajectories was below 3.5°, while the average *RMSE* of the knee joint angle trajectories remained under 5° across different scenarios and speeds, demonstrating the method’s high accuracy and effective personalization. In comparative tests with other models under identical experimental conditions, the proposed model achieved lower average *RMSE* with reduced error fluctuation, confirming its superior generalization capability. Regarding application validation, the proposed method—based on anthropometric features and the IGPR model—was successfully implemented on a lower-limb rehabilitation robot, enabling personalized gait generation and rehabilitation training for different patients under varying motion modes.

However, several limitations still exist in this study. First, the proposed method can only generate personalized gait trajectories offline based on static anthropometric features, target walking speed, and training scenarios. It cannot realize real-time gait adjustment or online adaptive correction according to the patient’s real-time motion intention, human-robot interaction force or gait deviation during rehabilitation training. Second, the model relies entirely on static anthropometric features as input, without integrating dynamic motion information such as surface electromyography (sEMG) signals, plantar pressure or joint velocity, which may limit its adaptability to stroke patients with severe motor dysfunction or abnormal gait compensation. Third, only two typical training scenarios (treadmill walking and overground walking) are included in the current research. In future work, more complex scenes such as stair ascent/descent and slope walking will be added to expand the dataset. Meanwhile, online learning, dynamic feedback correction or multi-source information fusion strategies will be introduced to realize real-time adaptive gait generation and improve the applicability of the model in clinical rehabilitation. Fourth, although the number of application validation subjects has been expanded to 4 to improve statistical power, all recruited subjects are healthy young people aged 25–31 years. Due to actual recruitment constraints, elderly individuals, patients, or people with significantly different body proportions were not included. Therefore, the generalization ability of the proposed method in elderly or stroke populations still needs to be further verified. To make the follow-up research more targeted and operable, we further define the quantitative standards for sample expansion and formulate a detailed clinical trial plan. In subsequent experiments, participants will be divided into three groups: healthy young adults aged 20–40 years, healthy elderly adults aged 60–75 years, and stroke hemiplegic patients. Each group will contain no fewer than 15 participants, covering different genders, body types, and patients with mild and moderate stroke. The follow-up clinical trials will be implemented in three stages. Firstly, we will collect gait data and anthropometric information from expanded participants to optimize and iterate the IGPR model. Secondly, comparative clinical experiments will be carried out to evaluate rehabilitation performance between the trajectories generated by our method and traditional fixed gait trajectories, with evaluation indicators including gait symmetry, joint activity range and human-robot coordination comfort. Finally, the model will be optimized continuously according to clinical feedback to form a complete set of personalized gait generation schemes for practical clinical rehabilitation scenarios.

In addition, although the model is trained on a healthy gait dataset, the proposed method focuses on personalized spatial-temporal gait structure rather than copying healthy gait patterns. Stroke patients exhibit pathological gait with compensatory movements, such as reduced joint range, uneven gait phase, and muscle weakness. However, the proposed method uses anthropometric features to adapt the spatial scale (joint amplitude) and temporal scale (gait period) according to individual physical conditions, which can provide a personalized basic reference trajectory. The generated trajectory does not fully replicate healthy gait but provides an individualized baseline that can be further corrected with clinical guidance. Apart from the above analysis, the current method still has evident limitations when dealing with patients suffering from severe pathological gait caused by hemiplegia. Typical symptoms of severe hemiplegia include severely limited range of motion on the affected limbs and long-term asymmetric compensatory gait formed during daily walking. Since the model is trained purely on healthy gait data, the generated baseline trajectory is designed according to normal human joint movement ranges. For patients with severe joint movement loss, the output trajectory may exceed the actual movable range of the affected limb. Moreover, the current model cannot actively identify or adapt to unilateral compensatory gait characteristics of hemiplegic patients. Therefore, this method is more applicable to patients with mild and moderate hemiplegia. For cases with severe pathological gait, it is necessary to integrate real-time joint angle feedback and compensatory gait recognition algorithms to optimize the generated trajectories in follow-up research.

This study presents a personalized gait trajectory generation method based on anthropometric features and an improved Gaussian process regression (IGPR) algorithm. By inputting 12 anthropometric features (such as height, weight, etc.) along with the target walking speed and rehabilitation scenario specified by a clinician into the IGPR model, the method directly generates highly adaptive gait trajectories tailored to individual patients. This effectively addresses the limitations of conventional gait generation methods, which often lack sufficient personalization and lead to poor compliance in human-robot coordinated motion control during gait rehabilitation training. Experimental results demonstrate that the proposed method reliably generates personalized gait trajectories for lower-limb rehabilitation robots across multiple walking speeds and various scenarios. In the future, this approach is expected to be applied in rehabilitation robotics to provide technical support for achieving human-robot motor coordination during gait training for patients with spinal cord injuries and stroke.

### Limitations of the study

This research has several obvious limitations. First, all training and validation datasets only include healthy young and elderly volunteers, without stroke hemiplegia patients with pathological compensatory gait. The model’s adaptability to abnormal clinical gait has not been verified. Second, the current framework only supports offline gait generation and lacks real-time online correction based on instantaneous gait deviation and human-machine interaction force. Third, the model only takes static body measurement data as input and does not integrate sEMG, plantar pressure and other dynamic physiological signals. Fourth, the experimental scenarios are limited to treadmill and flat walking, without stairs and slope walking tests. Fifth, only healthy volunteers completed robot tracking experiments, and controlled clinical rehabilitation trials are absent, so the therapeutic effect on stroke patients cannot be confirmed. Future research will expand subject groups and introduce real-time feedback algorithms to solve the above deficiencies.

## Resource availability

### Lead contact

Further information and requests for resources should be directed to and will be fulfilled by the lead contact, Huihui Sun (1112102005@zjut.edu.cn).

### Materials availability

This study did not generate new unique reagents.

### Data and code availability

• The datasets generated during this study are available at the Open Science Framework Repository: https://osf.io/krhju/(see key resources table).
• All original code are available at the Open Science Framework Repository: https://osf.io/krhju/ (see key resources table).
• Any additional information required to reanalyze the data reported in this study is available from the lead contact upon request.

## Acknowledgments

This work was financially supported by the Anhui Provincial Department of Education Scientific Research Project (grant no. 2025AHGXZK40330, grant no. 2025AHGXZK30553), the Anhui Province Science and Technology Innovation Break through Plan (no. 202423i08050056), the Anhui Provincial High-Level Talent Introduction and Cultivation Project - Young Top Talents (grant no. Wjgw2025313) and the 10.13039/501100002858China Postdoctoral Science Foundation (grant no. 2025M771716), the 10.13039/501100012166National Key R&D Program of China (grant no. 2022YFC3601702).

## Author contributions

Conceptualization, L.T. and H.S.; methodology, L.T. and H.S.; investigation, L.T., H.S., X.T., R.C., and Q.L.; formal analysis, L.T., H.S., and S.Z.; writing – original draft, L.T.; writing – review and editing, L.T. and H.S.; funding acquisition, L.T. and H.S.; resources, L.T., H.S., and S.Z.

## Declaration of interests

The authors declare no competing interests.

## STAR★Methods

### Key resources table

**Table:** 

REAGENT or RESOURCE	SOURCE	IDENTIFIER
**Deposited data**		

Public healthy human overground walking kinetics dataset	Horst et al.^26^; Mendeley Data	https://doi.org/10.17632/svx74xcrjr.2
Gait trajectory and anthropometric features dataset	This paper	https://osf.io/krhju/

**Software and algorithms**		

MATLAB R2020b	MathWorks	https://www.mathworks.com/products/matlab.html
OriginPro 2018	OriginLab	https://www.originlab.com/
Improved Gaussian Process Regression	This paper	https://osf.io/krhju/
Linear search hyperparameter optimization algorithm	This paper	https://osf.io/krhju/

**Other**		

Lower limb rehabilitation robot	Self-developed laboratory equipment	N/A
IMU sensor	Self-developed laboratory equipment	N/A

### Experimental model and study participant details

#### Participants

This study involved two groups of human subjects. The first group included 42 healthy adults from a public gait database (18 females and 24 males; 24 young adults aged 27.6 ± 4.4 years and 18 older adults aged 62.7 ± 8.0 years). The second group consisted of 4 healthy volunteers (all males, aged 25–31 years, mean age 27.5 ± 1.8 years) for the robot application validation. All participants were free from lower extremity motor dysfunction, musculoskeletal injury, neurological disorders, or gait abnormalities. All participants were of Asian ancestry (Han Chinese ethnicity).

#### Ethical approval and informed consent

This study was conducted in accordance with the principles of the Declaration of Helsinki. All participants provided written informed consent before enrollment in the study. This study has been approved by the Ethics Committee (Ethical Approval Number: KY2022-173).

#### Sex and gender influence

Both male and female participants were included in the dataset. Sex/gender was not identified as a significant confounding factor in this study, since the IGPR model takes gender as one of the 12 anthropometric input features and can automatically adapt to gender-related differences in gait patterns.

### Method details

This method is aimed at meeting the personalized gait trajectory generation requirements for lower limb rehabilitation robots. The overall flowchart of the personalized gait trajectory generation method for lower limb rehabilitation robots based on anthropometric features and the IGPR model proposed in this study is shown in Figure 3. First, this method designs an IGPR model based on a hybrid kernel function. By using this model, based on the database of anthropometric features and gait trajectories from healthy individuals, the mapping relationship between anthropometric features (*P*), desired walking speed (*V*), and training scenario (*G*) to both a normalized single-cycle gait trajectory and its corresponding gait period is established, and the model training is completed. Subsequently, by utilizing the trained IGPR model, the method takes as input the measured anthropometric features of each patient, along with the target walking speed and rehabilitation training scenario specified by a rehabilitation therapist, to generate a highly personalized gait trajectory that is optimally adapted to the each individual patient.

#### Data preprocessing

To obtain normalized single-cycle gait trajectories and corresponding gait period for each volunteer, the collected continuous walking gait data were segmented into multiple single-cycle gait trajectories. Since the segmented single-cycle gait trajectories exhibit varying periods, we applied a resampling technique^27^ to normalize all trajectories to a uniform gait cycle length. Subsequently, a normalized single-cycle gait trajectory was obtained by averaging the resampled trajectories. Furthermore, the average gait period calculated from all segmented single-cycle trajectories was selected as the standardized gait period for the normalized single-cycle gait trajectory. All regression procedures were performed based on this computed normalized gait trajectory and the average gait period. The final personalized gait trajectory is generated by temporally scaling the normalized gait trajectory using the resampling technique according to the generated average gait period. Taking the hip joint as an example, the overall preprocessing process is shown in Figure 4.

#### Anthropometric feature selection and importance analysis

The rationality and importance of anthropometric features directly affect the accuracy and computational efficiency of personalized gait trajectory generation. To avoid feature redundancy and ensure strong correlation with gait trajectories, the max-relevance and min-redundancy (mRMR) criterion is adopted for feature selection and importance evaluation.^28^ The optimization objectives are defined as follows: The first principle is to minimize the redundant information among anthropometric features; the second principle is to maximize the correlation between each feature and hip/knee joint angle trajectories.

Based on the mRMR principle, the importance score and ranking of the 12 anthropometric features are calculated quantitatively. Features with higher scores contribute more to gait trajectory prediction, while features with lower scores still retain certain auxiliary representation ability. The importance scores and rankings of all 12 anthropometric features are listed in Table 1.

It can be observed that the limb dimension features (e.g., distance of hip, thigh length, lower leg length) achieve the highest importance scores, which is consistent with the biomechanical mechanism that lower-limb skeleton size determines gait kinematics characteristics. Body morphology features (weight, height) also show strong correlation. Although several features including age, width of foot and width of ankle obtain relatively low importance scores, we retain all 12 anthropometric features in the final model. These low-ranking features carry subtle but unique individual physiological information. For stroke patients, age is closely correlated with gait rhythm and joint flexibility, while foot morphological parameters affect foot landing posture and overall gait stability. Removing these features will result in the loss of detailed individual characteristics and reduce the personalization performance of generated gait trajectories. In addition, the mRMR criterion has guaranteed minimal redundancy among all selected features. The extra computational overhead caused by retaining all features is negligible and will not affect the operational efficiency of the model on rehabilitation robots. Therefore, all 12 features are reserved to fully characterize individual differences.

#### Design of the IGPR algorithm for gait trajectory generation

Given the nonlinear relationship between the input parameters—anthropometric features (*P*), walking speed (*V*), and training scenario (*G*)—and the resulting gait trajectories, along with their approximately Gaussian distribution, Gaussian process regression (GPR) was selected as the regression framework. GPR is a system probability regression method based on non-parametric kernels, which is suitable for nonlinear regression fitting problems in datasets with small measurement noise.^29^^,^^30^ Therefore, this study constructs an improved Gaussian process regression (IGPR) model to generate personalized gait trajectories for lower-limb rehabilitation robots. The model takes as input 12 anthropometric features, the desired walking speed, and the training scenario. Its outputs are the normalized single-cycle gait trajectory and the standardized gait period, formally defined by (Equation 1).

(Equation 1) $$X=\left[\begin{array}{l} x_{1}\\ x_{2}\\ \vdots \\ x_{N} \end{array}\right]=\left[\begin{array}{cc} b_{1}^{T} & t_{1}\\ \vdots & \vdots \\ b_{1}^{T} & t_{T}\\ b_{2}^{T} & t_{1}\\ \vdots & \vdots \\ b_{N}^{T} & t_{T} \end{array}\right],Y=\left[\begin{array}{l} y_{1}\\ y_{2}\\ \vdots \\ y_{N} \end{array}\right]=\left[\begin{array}{l} \theta_{1}^{hip}\\ \vdots \\ \theta_{N}^{hip} \end{array}\begin{array}{l} \theta_{1}^{knee}\\ \vdots \\ \theta_{N}^{knee} \end{array}\begin{array}{l} \theta_{1}^{ankle}\\ \vdots \\ \theta_{N}^{ankle} \end{array}\begin{array}{l} \lambda_{1}\\ \vdots \\ \lambda_{N} \end{array}\right]$$

Where, *X* represents the input variables, *N* is the number of volunteers, and *b*_*N*_ = (*P*_*N*_, *V*_*N*_, *G*_*N*_) is the set of 12 anthropometric features, walking speed, and training scenario for the *N*-th subject. t is the time index within the normalized single-cycle gait trajectory, and *T* is the final index in the temporal framework. *Y* represents the output, comprising $\theta_{N}^{hip}$ (the normalized hip joint flexion-extension angle for the *N*-th subject), $\theta_{N}^{knee}$ (the normalized knee joint flexion-extension angle for the *N*-th subject), and *λ*_*N*_ (the standardized gait cycle duration for the *N*-th subject).

##### Design of the IGPR model

Gaussian process regression (GPR) is a non-parametric regression method based on Gaussian processes (GP), which can be fully described by a mean function *m*(*x*) and a covariance function *K*(*x*, *x*′). The choice of covariance function *K*(*x*, *x*′) is critical in determining the estimation accuracy of the GPR model.^31^^,^^32^^,^^33^ Conventional GPR algorithms, which typically employ a single kernel function as the covariance function, often struggle to adequately preserve complex data characteristics. This limitation leads to reduced estimation accuracy for multi-output variables and weaker overall model generalization, making it difficult to meet the requirements for accuracy and generalizability in personalized gait trajectory generation.

The core novelty of the proposed IGPR lies in the hybrid kernel function specifically designed for human gait characteristics. Human gait exhibits two typical features: 1) a smooth global trend of joint angle variation during a gait cycle; 2) significant periodicity caused by repeated swing and stance phases. Conventional single kernel functions cannot capture both features simultaneously. Therefore, this study proposes an improved Gaussian process regression (IGPR) model based on a hybrid kernel function to enhance the accuracy and generalizability of personalized gait trajectory generation. To better preserve data characteristics and improve the model’s generalization capability, a composite kernel function *K*_*CK*_(*x*, *x*′) was constructed as the covariance function for GPR. This hybrid kernel optimizes model performance across different feature scales through the combination of a rational quadratic kernel and a periodic kernel. The rational quadratic kernel is used to capture the global trend characteristics of gait trajectories, while the periodic kernel is adopted to describe local periodic variations in the gait pattern. The hybrid kernel function is defined as:

(Equation 2) $$\begin{array}{l} K_{CK}\left(x,x^{\prime }\right)=K_{RQ}\left(x,x^{\prime }\right)+K_{PE}\left(x,x^{\prime }\right)={\left(\sigma_{f}^{RQ}\right)}^{2}{\left[1+\frac{{\left(x-x^{\prime }\right)}^{T}M_{l}\left(x-x^{\prime }\right)}{2\alpha }\right]}^{-\alpha }+\\ {\left(\sigma_{f}^{PE}\right)}^{2}\;\exp\left(-2M_{l}\;\sin^{2}\left(\frac{\omega \left(x-x^{\prime }\right)}{2\pi }\right)\right) \end{array}$$

Where, *K*_*RQ*_ (*x*, *x*′) denotes the rational quadratic kernel and *K*_*PE*_ (*x*, *x*′) denotes the periodic kernel, *M*_*l*_ = *diag*[*l*^−2^] denotes a diagonal matrix of hyperparameters *l*, *l* is the length-scale parameter, it controls the overall smoothness of the global gait trend. A larger *l* produces a smoother fitted joint angle curve and suppresses local abrupt changes, while a smaller *l* can capture tiny fluctuations of the global trend but is more susceptible to data noise. $\theta =\left(\sigma_{f}^{RQ},l^{RQ},\alpha ,\sigma_{f}^{PE},l^{PE},\omega ,\sigma_{n}^{2}\right)$ is the set of hyperparameters, *α* is the scale factor that reflects the global variation amplitude of gait, which directly adjusts the overall variation range of lower-limb joint angles and matches the actual maximum flexion-extension amplitude of human joints during walking. *ω* is the periodic frequency parameter that determines the fluctuation period of gait, which governs the cycle of gait movement and accurately fits the repeated swing phase and stance phase within a single gait cycle. These hyperparameters jointly regulate the global trend and local periodic characteristics of the predicted gait trajectory, which fully explains the rationality of the proposed hybrid kernel for gait fitting.

Therefore, the Gaussian regression process for personalized gait trajectory generation can be expressed as follows:

Assuming the regression function *f*(*x*) = *ϕ*(*x*)^*T*^*ω* + *ε*,*ω* ∼ (0,∑_*p*_), the mean and variance of the Gaussian process are given by:

(Equation 3) m(x)=E[f(x)]=ϕ(x)E(ω)=0

(Equation 4) $$E\left[f\left(x\right)f\left(x^{\prime }\right)\right]=\phi {\left(x\right)}^{T}E\left(\omega \omega^{T}\right)\phi \left(x^{\prime }\right)=\phi {\left(x\right)}^{T}\sum_{p}\phi \left(x^{\prime }\right)$$

By definition, if *f*(*x*) and *f*(*x*′) jointly follow a Gaussian distribution *N*(0,*ϕ*(*x*)^*T*^∑_*p*_*ϕ*(*x*′)), then the covariance function between any two input vectors (*x*, *x*′) is given by:

(Equation 5) $$K\left(x,x^{\prime }\right)=\phi {\left(x\right)}^{T}\sum_{p}\phi \left(x^{\prime }\right)$$

For a dataset *D* = {*X*, *Y*} = {(*x*_*i*_, *y*_*i*_);*i* = 1,2, …,*N*}, if the prior distribution of the output vector *Y* is established as $Y∼N\left(0,K_{CK}\left(x,x\right)+\sigma_{n}^{2}I\right)$, then the joint Gaussian distribution of the output vector *y* and the predicted output value *y*∗ can be derived as:

(Equation 6) $$\left[\begin{array}{l} y\\ y^{*} \end{array}\right]∼N\left(0,\left[\begin{array}{cc} K_{CK}\left(x,x\right)+\sigma_{n}^{2}I & K_{CK}\left(x,x^{*}\right)\\ K_{CK}{\left(x,x^{*}\right)}^{T} & K_{CK}\left(x^{*},x^{*}\right) \end{array}\right]\right)$$

where the input vector *x*_*i*_ ∈ *X* consists of the 12 anthropometric features, desired walking speed, and training scenario, the output vector *y*_*i*_ ∈ *Y* comprises the normalized hip and knee joint flexion-extension angles and the gait period; $\sigma_{n}^{2}I$ represents the noise covariance matrix, $\sigma_{n}^{2}$ is the error variance, and *I* is the *n*-dimensional identity matrix.

Based on the above formulation, we obtain the probability distribution relating the input data *x*∗ (comprising anthropometric features *P*, desired walking speed *V*, and training scenario *G*) to the output *y*∗ (containing the normalized hip joint flexion-extension angle *θ*^*hip*^, knee joint flexion-extension angle *θ*^*knee*^, and gait period *λ*):

(Equation 7) $$p\left(y^{*}|X,Y,x^{*},\theta \right)=N\left(y^{*}|{\bar{y}}^{*},\text{cov}\left(y^{*}\right)\right)$$

where ${\bar{y}}^{*}=K_{CK}{\left(x,x^{*}\right)}^{T}{\left[K_{CK}\left(x,x\right)+\sigma_{n}^{2}I\right]}^{-1}$, $\sigma_{n}^{2}I_{n}$ represents the noise covariance matrix, *I*_*n*_ is the *n*-dimensional identity matrix, and ${\bar{y}}^{*}$ is the estimated mean of *y*∗, cov(*y*∗) denotes the estimated variance, $\text{cov}\left(y^{*}\right)=K_{CK}\left(x^{*},x^{*}\right)-K_{CK}{\left(x,x^{*}\right)}^{T}{\left[K_{CK}\left(x,x\right)+\sigma_{n}^{2}I_{n}\right]}^{-1}$_._

##### Hyperparameter optimization of the IGPR model

The IGPR algorithm designed in this study is defined by the aforementioned mean function *m*(*x*) and the mixed kernel function *K*_*CK*_(*x*, *x*), and its characteristics are adjusted through the hyperparameters set $\theta =\left(\sigma_{f}^{RQ},l^{RQ},\alpha ,\sigma_{f}^{PE},l^{PE},\omega ,\sigma_{n}^{2}\right)$. Since the generalization performance of the IGPR model is influenced by the selection of its hyperparameter values, optimizing these hyperparameters is essential. Usually, the maximum likelihood method is used to solve it, and the objective function is to maximize the log likelihood function. However, it is difficult to overcome the shortcomings of strong dependence on initial values and uncertain number of iterations.

Overcome these limitations, this study employs a linear search algorithm to optimize each hyperparameter in the set.^34^ As verified in section results, compared with maximum likelihood estimation (MLE) and Bayesian optimization, the linear search method shows better stability, faster convergence, lower computational cost, and less sensitivity to initial values, making it more suitable for hyperparameter optimization in the personalized gait trajectory generation task.

#### Implementation of the IGPR model

The overall execution workflow of the IGPR model is depicted in Figure 5, with the main steps outlined as follows:

Step 1: Data acquisition and preprocessing. Raw kinetic and whole-body kinematic data during walking were collected from a diverse participant to construct an anthropometric features database and a gait trajectory database. The data were then preprocessed and partitioned into datasets.
Step 2: IGPR model construction and parameter optimization. Initialize the IGPR parameters and determine the IGPR prior model $y∼N\left(0,K_{CK}\left(x,x\right)+\sigma_{n}^{2}I\right)$. Use a linear search algorithm to optimize the hyperparameter set *θ* of the IGPR covariance function, output the optimal hyperparameters to construct the optimal IGPR model, and establish the joint Gaussian distribution.
Step 3: Personalized gait trajectory generation. The testing set data is input into the established optimal IGPR model to predict both the normalized single-cycle gait trajectory and the gait period, thereby generating the personalized gait trajectory.

#### Experimental validation

To train and test the proposed IGPR model and validate the effectiveness of the presented method, the following experimental study was conducted.

This experimental study utilized a publicly available gait kinematics database of healthy adults.^26^ The database comprises raw kinetic and whole-body kinematic data from 42 healthy subjects (18 female, 24 male), including 24 younger adults (age 27.6 ± 4.4 years, height 171.1 ± 10.5 cm, weight 68.4 ± 12.2 kg) and 18 older adults (age 62.7 ± 8.0 years, height 161.8 ± 9.5 cm, weight 66.9 ± 10.1 kg). It contains each subject’s age, height, weight, and raw kinetic/whole-body kinematic data recorded during walking under multiple speeds across two training scenarios (treadmill walking and overground walking). All participants were free of gait pathologies and lower-limb pain or injury.

The 12 selected anthropometric features in this study include gender, age, height, weight, and 8 body shape characteristics (as shown in Figure 6). Among them, the data of the 8 body shape characteristics can be calculated based on the static calibration data collected in the database. This study incorporated two training scenarios: “treadmill walking” and “overground walking.” For treadmill walking, three speed conditions were selected: 0.4*V* m/s, 0.7*V* m/s, and 1.0*V* m/s, where *V* represents the subject’s normal walking speed on a treadmill. For overground walking, two speed conditions were chosen: *C* m/s and *S* m/s, where *C* m/s denotes the subject’s self-selected comfortable walking speed, and *S* m/s indicates a slow walking speed overground. The kinematic data for all specified speed conditions are available in the database.^26^ The corresponding normalized single-cycle gait trajectories and gait periods for each subject under the different training scenarios and walking speeds can be calculated from this kinematic data.

To further validate the feasibility of the proposed method, we recruited 4 healthy subjects (age 27.6 ± 4.4 years, height 171.1 ± 10.5 cm, weight 68.4 ± 12.2 kg) and conducted an applied validation using the trained IGPR model and a self-developed flexible lower-limb gait rehabilitation robotic platform. In this experiment, the subject’s left leg was defined as the paretic leg requiring assisted rehabilitation, while the right leg was designated as the healthy leg. The personalized gait trajectories required for the 4 subjects during rehabilitation training were generated by inputting manually measured anthropometric features (including age, height, and weight) along with the specified training scenario and desired walking speed into the trained IGPR model. Meanwhile, the actual hip and knee joint angle trajectories of the paretic leg during training were obtained using inertial measurement units (IMUs) independently developed by our research group. In this experiment, three IMU sensors were employed to capture these trajectories, with the specific sensor placement scheme illustrated in Figure 7.

**Figure 13:**
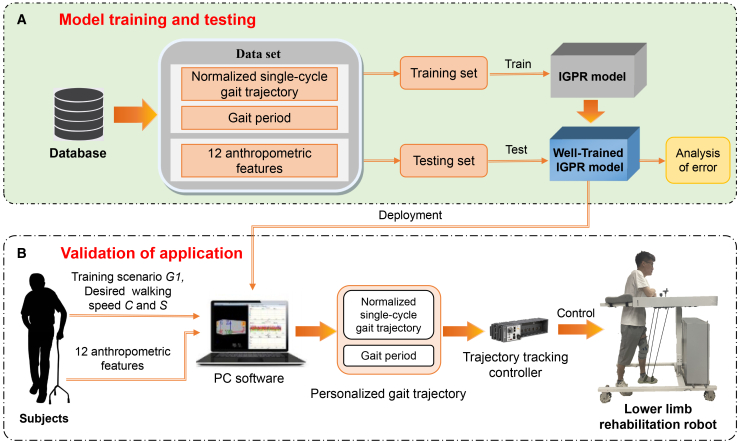
Overall experimental flowchart (A) IGPR model training and testing process. (B) Application validation process.

The specific experimental protocol was designed as follows:

① IGPR model training and testing protocol: First, the 12 anthropometric features for each subject in the public database^26^ were extracted and calculated, along with the normalized single-cycle gait trajectories and gait periods corresponding to five walking speeds (0.4*V* m/s, 0.7*V* m/s, 1.0*V* m/s, *C* m/s, and *S* m/s) under both treadmill and overground walking scenarios. These data were used to construct the dataset. The specific 12 anthropometric features required are illustrated in Figure 6. Next, the constructed dataset was partitioned, with data from 38 subjects forming the training set Gtrain, and data from the remaining four subjects comprising the test set Gtest. Finally, based on the partitioned datasets, the IGPR model was trained and tested, followed by an error analysis of the test results. The overall experimental workflow is shown in Figure 13A.
② Application validation protocol: First, 4 healthy subjects were recruited, and their 12 anthropometric features were measured. These parameters were then input into the trained IGPR model via the host computer to generate personalized gait trajectories (including the normalized single-cycle gait trajectory and gait period) for each subject walking on level ground at speeds of *S* m/s and *C* m/s, respectively. Next, the subjects were assisted in properly donning the self-developed flexible lower-limb gait rehabilitation robot, and IMU sensors were attached according to the predefined placement scheme. Finally, the generated personalized gait trajectories were sequentially sent to the robot controller for trajectory tracking. Tracking errors under different conditions were subsequently analyzed. The overall experimental procedure is illustrated in Figure 13B.

### Quantification and statistical analysis

Four evaluation metrics are adopted: root-mean-square error (*RMSE*), mean absolute error (*AE*), coefficient of determination (*R*^2^). Baseline comparison models include random forest (RF) and traditional single-kernel GPR. Dataset split rule: 38 subjects as training set, 4 subjects as test set. All experimental results are expressed as mean ± standard deviation (SD), and all statistical data are presented in Tables 2, 3, 4, 5, 6, 7, 8, and 9 and Figures 1, 2, 8, 9, 10, 11, and 12. Linear search algorithm is used for IGPR hyperparameter optimization, compared with MLE and Bayesian optimization. No inter-group significance test is carried out in this work; only prediction precision is quantified. All data processing, feature screening and statistical calculations were implemented in MATLAB R2020b, with figures and tables generated by Origin 2018.
